# Effect of cognitive emotional al social participation status over the emotional state and learning of new information in older adults who attend a museum of science visit: preliminary results

**DOI:** 10.1002/alz70860_100485

**Published:** 2025-12-23

**Authors:** Carolina Feldberg, Irrazabal Natalia, Silvia Deborah Ofman, Rosario Quian, Fernando Tonini, Mercedes Conti Urabayen, Constanza Gosci, Florencia Spellanzon, Maria Jose Ruiz

**Affiliations:** ^1^ Conicet‐ Ineba, Buenos Aires, Argentina; ^2^ Universidad de Palermo, Buenos Aires, Argentina; ^3^ Ineba ‐ Conicet, CABA, Argentina; ^4^ CONICET‐INEBA, caba, Argentina; ^5^ Universidad de Palermo, caba, Ciudad Autónoma de Buenos Aires, Argentina; ^6^ Universidad de Buenos Aires Facultad de Psicologia, caba, Ciudad Autónoma de Buenos Aires, Argentina

## Abstract

**Background:**

Cultural and educational activities have been shown to have a positive impact on different areas related to the physical, emotional, cognitive and social well‐being of older adults. In this context, cultural organizations can play a complementary role to that of health institutions. The role of museums is highlighted as they provide opportunities for social interaction, promote relaxing learning experiences and the acquisition of new skills, which lead to greater self‐esteem, a sense of identity, inspiration, as well as reducing social isolation and anxiety. However, access visits to museums are relatively new topics in the field of health and it is relevant to study their recreational, educational and potential health‐promoting impact on older adults. The present study aims to analyze whether social participation and prior cognitive and mood state influence learning and emotional state after visiting a science museum with a population of self‐sufficient older adults.

**Method:**

200 older participants (M = 71.87 years; SD = 6.46 years) made a 30‐minute visit to a science museum in Buenos Aires, Argentina. The following were administered: Social Participation Questionnaire, MOCA, Geriatric Depression Scale (GDS), Generalized Anxiety Disorder 7‐item (GAD‐7) Questionnaire for learning new information and PANAS‐State Scale.

**Result:**

The results obtained indicate that higher levels of social participation are positively associated with the presence of greater positive emotions (*r* = .22; *p* < .01) and inversely with negative emotions (‐.135; *p* < .05) after the visit. In addition, it is observed that those subjects who have greater general participation in their daily life present lower indicators of depression (*r* = .‐208; *p* < .01) and anxiety (*r* = .‐131; *p* < .05). In turn, those who have a better cognitive status evaluated through the MOCA test learn more new information during the visit to the museum (.501; *p* < .01).

**Conclusion:**

These results indicate that leisure activities could play a differential and important role in the construction of cognitive reserve throughout the life cycle, constituting a possible cognitive protection factor in the aging process and an effective intervention tool to regulate the emotional state in older adults.

